# Karyotype characteristics and chromosomal polymorphism of *Chironomus* “annularius” sensu Strenzke (1959) (Diptera, Chironomidae) from the Caucasus region

**DOI:** 10.3897/CompCytogen.v12i3.25832

**Published:** 2018-07-30

**Authors:** Mukhamed Kh. Karmokov

**Affiliations:** 1 Tembotov Institute of Ecology of Mountain territories RAS, I. Armand str., 37a, Nalchik 360051, Russia Tembotov Institute of Ecology of Mountain territories, Russian Academy of Sciences Nalchik Russia

**Keywords:** Diptera, Chironomidae, *
Chironomus annularius
*, polytene chromosomes, chromosome polymorphism, Central Caucasus, South Caucasus, Eastern Ciscaucasia

## Abstract

The study presents data on the karyotype characteristics and features of chromosomal polymorphism of *Chironomus* “annularius” sensu Strenzke (1959) (Diptera, Chironomidae) from three populations of the Caucasus region (South and Central Caucasus, and Eastern Ciscaucasia). We found 17 banding sequences in the Caucasian populations. We observed inversion polymorphism in almost all chromosome arms except for arm G. The genetic distances between all the studied populations of *Ch.* “annularius” were calculated using Nei criteria (1972). In spite of relative geographic proximity, the genetic distances between populations of the Caucasus are quite large, and they do not form a single cluster of Caucasian populations. The population of the South Caucasus goes to the European cluster, the population of the Central Caucasus goes to the Asian cluster and the population of Eastern Ciscaucasia does not belong to any of the outlined clusters. Principal component analysis (PCA) shows a similar picture. Two of the Caucasian populations do not follow Hardy-Weinberg expectation, there being a marked deficiency of heterozygotes in arms A, B and C, arguably, due to negative selection of heterozygotes or founder effect. All the obtained data are indicative of the complex genetic structure of Caucasian populations of *Ch.* “annularius” and total complexity microevolution processes occurring in the Caucasus region.

## Introduction

There are a great number of publications that mention the name of *Chironomusannularius* from the 18^th^ century (Spies and Sæther 2004). According to Spies and Sæther there are several different species under this name and revision of the species described under the *Ch.annularius* name is necessary. The most complete descriptions of *Ch.annularius* morphology and karyotype were presented by Strenzke (1959), Keyl and Keyl (1959), Keyl (1962). For this reason, Spies and Sæther (2004) suggest using the name *Ch.* “annularius” sensu Strenzke (1959) until revision completion. According to the Fauna Europaea web source (http://www.faunaeur.org) the species is common in Western (British Isles, Norway, Sweden, Finland, French mainland, Germany, Spanish mainland, Italian mainland and so on) and Eastern Europe (Poland, Romania, Bulgaria, Ukraine and so on). Also, according to Kiknadze et al. (2016), the species is known from European Russia, the Ural, Western Siberia, the Republics of Altai, Tuva, and Sakha (Yakutia), Kazakhstan, the USA (several sites) and Canada (Alberta, Amisk Lake).

Keyl & Keyl (1959) described the karyotype of *Ch.* “annularius” sensu Strenzke (1959) from German populations. At first, Keyl (1962) and Kiknadze et al. (1991a) mapped chromosome arms A, E and F. Later, Kiknadze et al. (1996c, 2012) mapped arms C and D. Belyanina (1981), Petrova and Michailova (1986) presented some information on karyotype and chromosomal polymorphism of Palearctic *Ch.* “annularius” populations using an arbitrary system of chromosome mapping or without any mapping (Michailova 1989). The karyotype and chromosomal polymorphism of *Ch.* “annularius” from Nearctic populations were studied relatively later than that from Palearctic populations (Butler et al. 1995, Andreeva 1999, Kiknadze et al. 2008b, 2010, 2012).

Karmokov (2012) previously briefly described the karyotype and chromosomal polymorphism of *Ch.* “annularius” from one Central Caucasian population.

The aim of the work was to present the description of karyotype characteristics and chromosomal polymorphism of *Ch.* “annularius” from three Caucasian populations. In addition, it was also very important to compare the chromosomal polymorphism characteristics of *Ch.* “annularius” from the Caucasus with earlier studies.

## Methods

**Figure 1.**
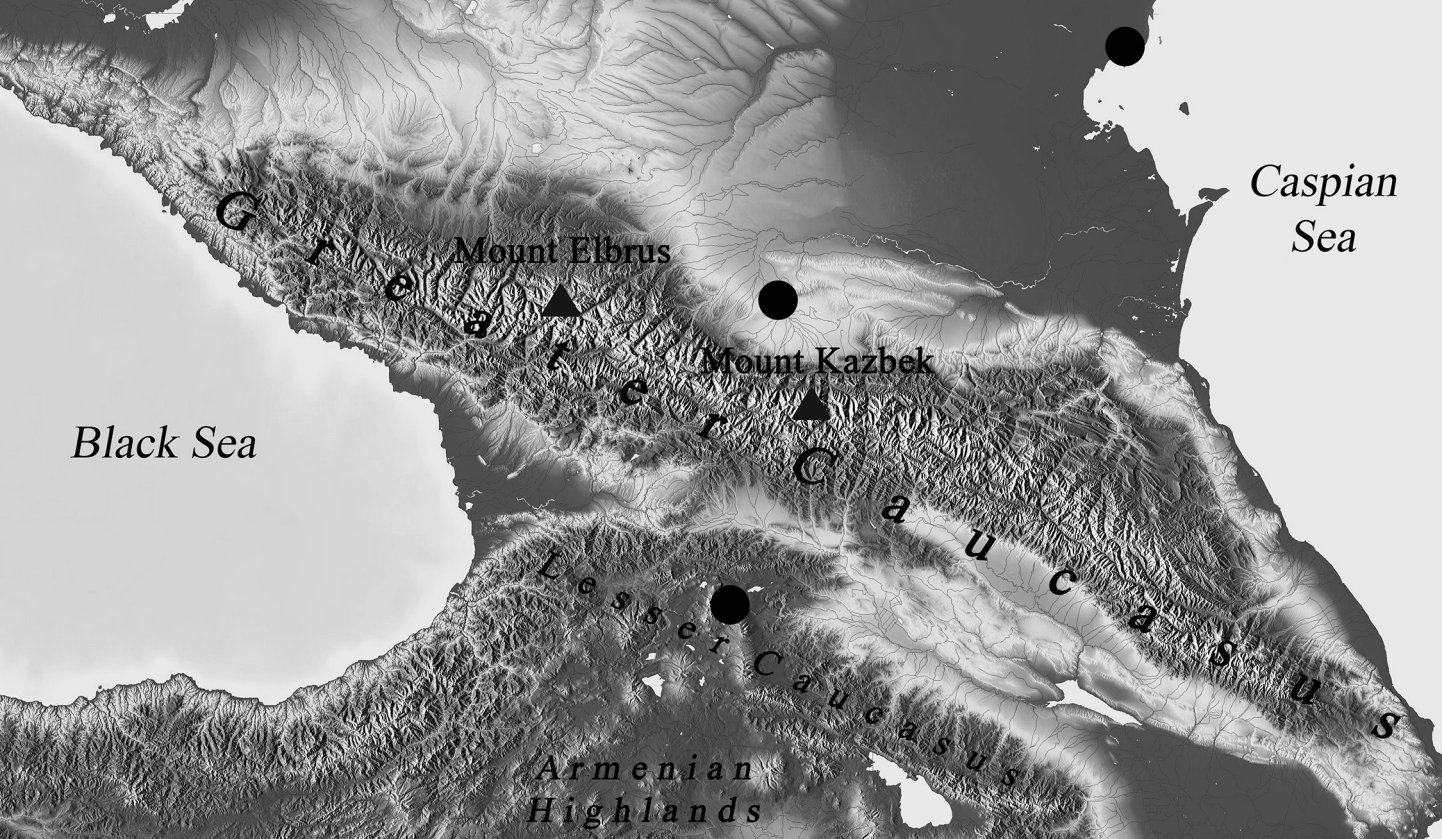
Collections sites of *Ch.* “annularius” in Caucasus region. Collection sites are marked with black dots.

We used fourth instar larvae of *Chironomus* in the karyological study. We provide the collection sites and abbreviations of earlier studied populations (Kiknadze et al. 2012) in Table 1. The Caucasus region served as larval collection sites and included one site from Republic of North-Ossetia-Alania (Russian Federation), one site from the Republic of Dagestan (Russian Federation) and one site from the Republic of Georgia (Table 2). Collection sites are marked on the map with dark dots (Fig. 1). The geographic division of the Caucasus follows Gvozdetskii (1963). The area to the west of Mount Elbrus considered as the West Caucasus. The area between Mount Elbrus and Mount Kazbek considered as the Central Caucasus, and the area to the east of Mount Kazbek as the East Caucasus. The area, including the Kuban-Azov Lowland in the west, the Stavropol Upland in the middle and the Terek-Kuma Lowland in the east considered as Ciscaucasia. The area, including the Colchis Lowland, the Kura-Aras Lowland, the Lesser Caucasus, the Talysh mountains, the Lenkoran Lowland and eastern portion of the Armenian Highlands considered as the South Caucasus or Transcaucasia.

**Table 1.** Collection sites and number of analyzed *Ch.* “annularius” larvae from the European, Siberian, Kazakhstan and Nearctic populations per Kiknadze et al. (2012).

Localities	Population abbreviation	Collection sites	Collection date	Number of specimens
European population	NL-NT-NT	The Netherlands	07.1998	16
Siberian populations	RU-OMS-IR	Omskaya Oblast’: former riverbed or river Irtysh near Omsk	08.1996	39
	RU-NSK-EP	Reservoir near river Nizhnyaya Eltsovka	07.2006	26
	RU-NSK-BE	Pond in Berdsk	06.1998	52
Kazakhstan population	KZ-SIP-UB	Alma Ata, pond in the Botanical garden	09.1989	17
Nearctic populations	US-ND-WA	USA, Warsing Dam	09.05.96	16
	US-ND-IS	USA, Isabel Lake	02.1995	33

**Table 2.** Collection sites and number of analyzed *Ch.* “annularius” larvae from the Caucasus region.

Localities	Population abbreviation	Collection sites	Collection date	Number of specimens
Central Caucasus	CC-OS-ZM	43°19.9067’ N; 44°11.1333’ E, Republic of North-Ossetia-Alania, puddle in the bed of drained pond, beside the Zmeiskaya settlement, altitude ca 310 m a.s.l.	05.05.10	32
Eastern Ciscaucasia	ECS-BK-ART	44°45.965’ N; 46°48.2037’ E, Republic of Dagestan, Tarumovsky District, ca 8 km southwest of “Biriuziak” holyday base, a puddle beside the artesian well, altitude ca -25 m b.s.l.	26.05.17	47
South Caucasus	SC-SJ-PA	41°19.3018’ N; 43°45.5577’ E, Republic of Georgia, Samtskhe-Javakheti region, ca 1 km north to the Sagamo settlement, one of branches of the Paravani river, altitude ca 2010 m a.s.l.	18.07.17	36

Consequently, the site from Republic of North-Ossetia-Alania belongs to the Central Caucasus, the site from the Republic of Dagestan belongs to the Eastern Ciscaucasia and the site from the Republic of Georgia belongs to South Caucasus or Transcaucasia. Regarding vertical zonation (Sokolov and Tembotov 1989), the first site belongs to the Terek variant, the second site belongs to the semi-steppe zone and the last one to the Javakheti-Armenian variant.

The head capsule and body of 25 larvae were slide mounted in Fora-Berlese solution. The specimens have been deposited in the Tembotov Institute of Ecology of Mountain territories RAS in Nalchik, Russia. We studied the karyotype and chromosomal polymorphism in 115 larvae from the Caucasus region.

We fixed the larvae for karyological study in ethanol-glacial acetic acid solution (3:1). The slides of the chromosomes were prepared using the ethanol-orcein technique (see Dyomin and Ilyinskaya 1988, Dyomin and Shobanov 1990). The banding sequences were designated per the accepted convention specifying the abbreviated name of the species, symbol of chromosome arm, and sequence number as in annA1, annA2, etc. (Keyl 1962, Wülker and Klötzli 1973).

We performed the identification of chromosome banding sequences for arms A, E and F using the photomaps of Kiknadze et al. (2012, 2016) in the system of Keyl (1962) and chromosome mapping for arms C and D as per Kiknadze et al. (1996c, 2012) in the system of Dévai et al. (1989).

We studied the chromosome slides using a Carl Zeiss Axio Imager A2 microscope and performed the statistical data processing using software packages PAST 3.18 (Hammer et al. 2001), GenALEx 6.503 (Peakall and Smouse 2006, 2012) and STATISTICA 10 (StatSoft).

We used the following parameters of chromosomal polymorphism characteristics for comparison: percentage of heterozygous larvae, number of heterozygous inversions per larvae, the number of banding sequences in a population and a number of genotypic combinations per population. We calculated the genetic distances between populations according to Nei criteria (Nei 1972) using Chironomus 1.0 software (Kazakov and Karmokov 2015) based on original data along with Kiknadze et al. (2012) research results.

We used the software package GenALEx 6.503 (Peakall and Smouse 2006, 2012) to check if the Caucasian populations follow Hardy-Weinberg expectation.

We performed a principal component analysis (PCA) of all the studied populations using original and previous data of Kiknadze et al. (1996c, 2012) to obtain a broader overview of the population genetic relationships (Fig. 5).

We measured the genetic distances (Table 6) between populations by Nei criteria (1972) based on original and previous data of Kiknadze et al. (1996c, 2012). Also, we constructed the tree dendrogram of genetic distances of studied populations using single-linkage clustering based on the obtained values (Fig. 6).

## Results

We attributed the larvae of *Chironomus* in the studied sites to *Ch.* “annularius” by both morphological and chromosomal characteristics. The morphological larval characters of *Ch.* “annularius” from the Caucasian sites are similar to those previously described for this species by Kiknadze et al. (1996c, 2012).

### Karyotype of *Ch.* “annularius” from the Caucasus region

**Figure 2.**
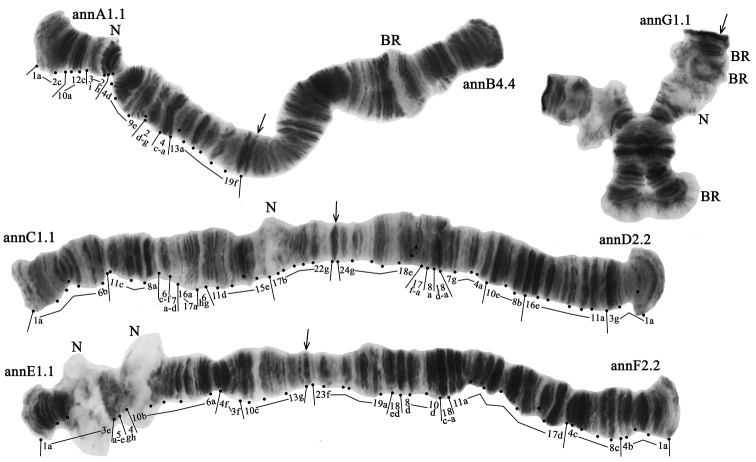
Karyotype of *Ch.* “annularius” from the Caucasus region; annA1.1, annD2.2 etc. – genotypic combinations of banding sequences; BR – Balbiani rings, N – nucleolus. Arrows indicate centromeric bands.

The diploid number of chromosomes in *Ch.* “annularius” karyotype is 2n = 8, chromosome arm combination is AB, CD, EF, and G (the “thummi” cytocomplex) (Fig. 2). Chromosomes AB and CD are metacentric, EF is submetacentric, and G is telocentric. There are four permanent nucleoli (N) in karyotype: one nucleolus in arm C, two in the arm E and one in arm G. Besides permanent nucleoli there is a fluctuating nucleolus on arm A (region 2d-3a) that can be detected in most larvae of previously studied populations in homo- or heterozygous state (Kiknadze et al. 2012). The nucleolus on arm A is present in all the Caucasian populations (Fig. 2). There are four Balbiani rings (BR) in the karyotype: three in arm G and one in arm B (Fig. 2).

### Banding sequences and chromosomal polymorphism of *Ch.* “annularius” from the Caucasus region.

Previously, Kiknadze et al. (2012) described 24 banding sequences in *Ch.* “annularius” banding sequences pool. In the studied populations, 15 of those sequences are present, and two banding sequences have been found for the first time, providing 17 banding sequences in the Caucasian populations (Table 3).

**Figure 3.**
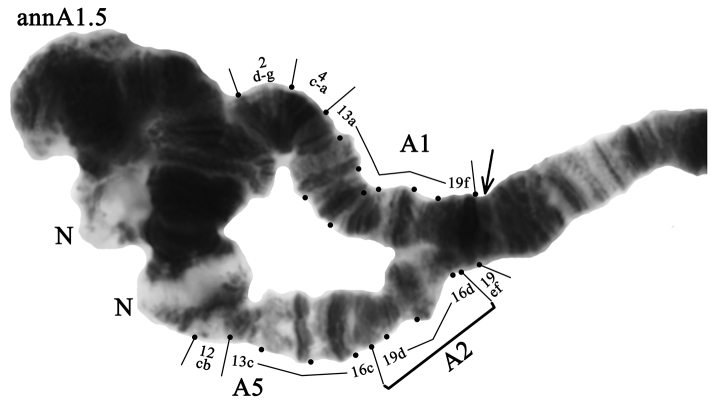
Heterozygous genotypic combination annA1.5. Designations as in Fig. 2.

**Arm A** has four banding sequences: annA1, annA2, annA3, and annA5 (Figs 2–3, Table 3). The banding sequence annA1 and genotypic combination annA1.1 were predominant in populations of Eastern Ciscaucasia and South Caucasus (Tables 3, 4). In population of Central Caucasus, the banding sequence annA2 and genotypic combination annA2.2 were predominant. The banding sequence annA5 is new for the species and described for the first time (Fig. 3, Tables 3, 4). It differs from annA2 by one simple inversion step that involves regions 16d-19d:

annA5 1a-2c 10a-12a 13ba 4a-c 2g-d 9e-4d 2h-3i 12cb 13c-16c 19d-16d 19ef C

The banding sequence annA5 was found only in the population of the South Caucasus with relatively low frequency (annA5 – 0.069) and only in the heterozygous state (annA1.5 – 0.139) (Tables 3, 4).

**Arm B** has three banding sequences: annB1, annB2, and annB4 (Fig. 2; Tables 3, 4). The banding sequence annB1 and genotypic combination annB1.1 were predominant in the population of South Caucasus (Tables 3, 4). The banding sequence annB2 and genotypic combination annB2.2 were predominant in the population of Eastern Ciscaucasia. The banding sequence annB4 and genotypic combination annB4.4 were dominant in the population of Central Caucasus.

**Arm C** has two banding sequences: annC1 and annC2 (Fig. 2). The banding sequence annC1 and genotypic combination annC1.1 were predominant in populations of Central and South Caucasus (Tables 3, 4). In the population of Eastern Ciscaucasia, the banding sequence annC2 and genotypic combination annC2.2 were predominant.

**Figure 4.**
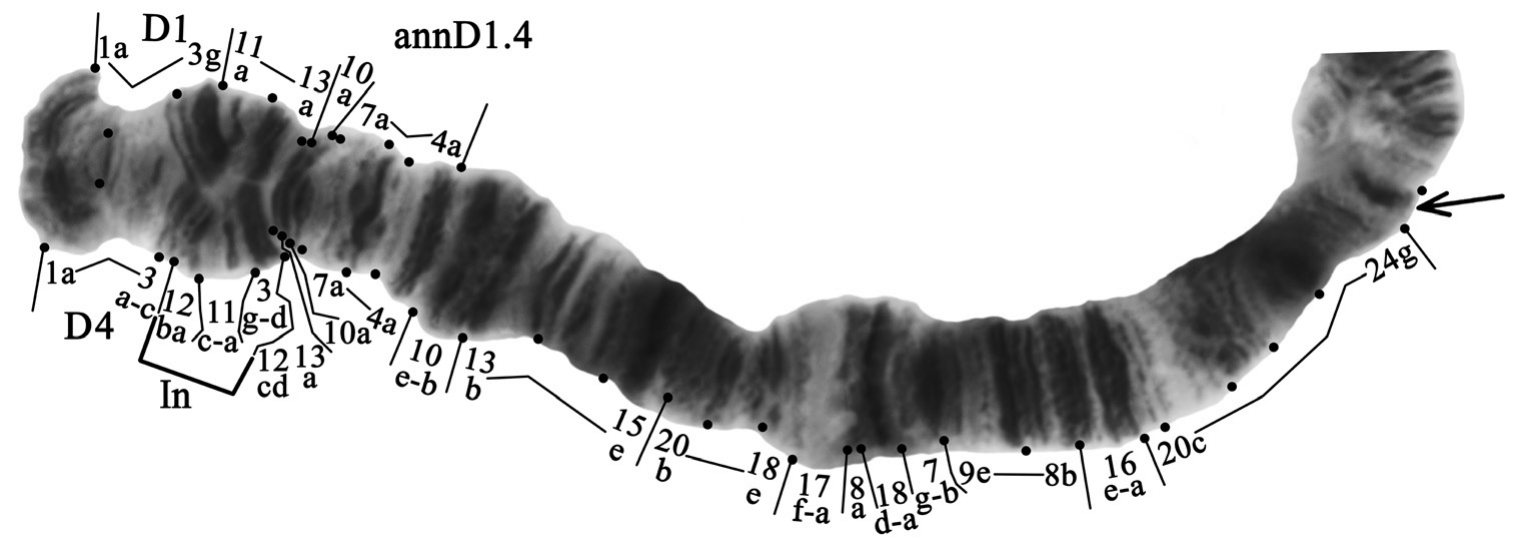
Heterozygous genotypic combination annD1.4. Designations as in Fig. 2.

**Arm D** has three banding sequences: annD1, annD2, and annD4 (Fig. 2). The banding sequence annD1 and genotypic combination annD1.1 were predominant in populations of Central and South Caucasus (Tables 3, 4). In the population of Eastern Ciscaucasia, the banding sequence annD2 and genotypic combination annD2.2 were predominant. The banding sequence annD4 is new for the species and described for the first time (Fig. 4, Tables 3, 4). It differs from annD1 by one simple inversion step that involves regions 3d-g 11a-c 12ab:

annD4 1a-3a-c 12ba 11c-a 3g-d 12dc 13a 10a 7a-4a 10e-b 13b-15e 20b-18e 17f-a 8a 18d-a 7g-b 9e-8b 16e-a 20c-24g C

The banding sequence annD4 was found only in the population of the South Caucasus with very low frequency (annD4 – 0.014) and only in the heterozygous state (annD1.4 – 0.028) (Tables 3, 4).

**Arm E** has two banding sequences: annE1 and annE2 (Fig. 2). The banding sequence annE1 and genotypic combination annE1.1 were predominant in populations of Central and South Caucasus (Tables 3, 4). In the population of Eastern Ciscaucasia, the banding sequence annE2 and genotypic combination annE2.2 were predominant.

**Arm F** has two banding sequences: annF1 and annF2 (Fig. 2). The banding sequence annF2 and genotypic combination annF2.2 were predominant in populations of Central and South Caucasus (Tables 3, 4). In the population of Eastern Ciscaucasia the banding sequence annF1 and genotypic combination annF1.1 were predominant.

**Arm G** was monomorphic with banding sequence annG1.1 (Fig. 2, Tables 3, 4).

### Comparison of chromosomal polymorphism of *Ch.* “annularius” from the Caucasian populations with that of populations of other regions.

The data for European (Netherlands), Siberian, Kazakhstan and Nearctic (USA) populations are available due to Kiknadze et al. (1996c, 2012).

**Arm A.** Most earlier studied populations (Kiknadze et al. 1996c, 2012) were characterized by the presence of three banding sequences annA1, annA2 and annA3 (Table 3). The same picture was observed in the population of Eastern Ciscaucasia. In the European and Central Caucasian populations, two sequences were found, annA1 and annA2. In the Nearctic population of Warsing Dam (US-ND-WA) only sequence annA2 was present and in the second Nearctic population sequences annA2 and annA4 were present (Table 3). The most polymorphic population for this arm was the population of the South Caucasus, where four sequences (annA1, annA2, annA3 and annA5) and six genotypic combinations (annA1.1, annA1.2, annA1.3, annA1.5, annA2.2 and annA2.3) were present (Table 4). The banding sequence annA5 might be endemic for the region of South Caucasus (Table 3). In all the studied populations, sequences annA3, annA4 and A5 has been observed only in the heterozygote state (Table 4).

**Arm B** is polymorphic in most parts of studied populations, except for the Nearctic population of Warsing Dam where only banding sequence annB2 was present and the population of the Central Caucasus where also only sequence annB4 was found (Table 3). In European and Siberian populations, two sequences annB1 and annB2 were present, while in the first population sequence annB2 has been observed only in the heterozygote state (Table 4). In populations of Europe and South Caucasus, genotypic combination annB1.1 was predominant. The banding sequence annB2 in the homozygous state was predominant in populations of Eastern Ciscaucasia, Siberia and Nearctic population of Isabel Lake (US-ND-IS), while in the Kazakhstan population heterozygote annB2.4 was predominant (Tables 3, 4).

**Arm C** of *Ch.* “annularius” is polymorphic in two Caucasian populations (Eastern Ciscaucasia and Central Caucasus), Kazakhstan population and one Siberian population (Pond in Berdsk). The arm is monomorphic in populations of Europe, South Caucasus and rest of the Siberian populations, where only genotypic combinations annC1.1 was present. In addition, the arm is monomorphic in both Nearctic populations where the other genotypic combination annC3.3 was found (Table 4). In the population of the Eastern Ciscaucasia banding sequences annC1 and annC2 were present in both homozygous and heterozygous state with predominance of genotypic combination annC2.2 (Tables 3, 4). A similar picture observed in the population of Kazakhstan, where also both sequences annC1 and annC2 were found, but sequence annC1 was present only in heterozygous state and genotypic combination annC2.2 was dominant.

**Arm D** of *Ch.* “annularius” is polymorphic in most of the studied populations, except for both Nearctic populations, where only banding sequence annD3 was present and population of the Europe where only sequence annD1 was found (Table 3). In populations of Siberia and the population of South Caucasus genotypic combination annD1.1 was predominant, while in Kazakhstan population heterozygous combination annD1.2 was predominant. The banding sequence annD4 is probably endemic for the region of South Caucasus (Table 3). In populations of Eastern Ciscaucasia and Central Caucasus, two banding sequences annD1 and annD2 were found with predominance of genotypic combination annD2.2. The banding sequence annD1 in these populations was found only in the heterozygous state (Tables 3, 4).

**Arm E** of *Ch.* “annularius” is polymorphic in most part of the studied populations, except for the Nearctic population of Warsing Dam and the population of the Europe where only banding sequence annE1 was present (Table 3). In the second Nearctic population, Kazakhstan population and two Caucasian populations (Central and South Caucasus) banding sequences annE1 and annE2 are presented in both homozygous and heterozygous state with predominance of genotypic combination annE1.1. A similar picture is observed in Siberian populations, but here another combination annE1.2 was predominant (Tables 3, 4). In the population of Eastern Ciscaucasia unlike all other populations the genotypic combination annE2.2 was predominant (Table 4).

**Table 3.** Frequency of banding sequences in different populations of *Ch.* “annularius”. N – the number of individuals, * – original data.

Banding sequences	European population	Caucasian populations			Siberian populations			Kazakhstan population	Nearctic populations	
	NL-NT-NT N=16	ECS-BK-ART N=47*	CC-OS-ZM N=32*	SC-SJ- PA N=36*	RU-OMS-IR N=39	RU-NSK-EP N=26	RU-NSK-BE N=52	KZ-AA-BG N=17	US-ND-WA N=16	US-ND-IS N=33
A1	0.438	0.766	0.313	0.708	0.910	0.769	0.865	0.736	0	0
A2	0.562	0.074	0.687	0.181	0.052	0.212	0.096	0.235	1	0.985
A3	0	0.160	0	0.042	0.038	0.019	0.039	0.029	0	0
A4	0	0	0	0	0	0	0	0	0	0.015
A5	0	0	0	0.069	0	0	0	0	0	0
B1	0.844	0	0	0.778	0.051	0.173	0.106	0	0	0
B2	0.156	0.596	0	0	0.949	0.827	0.894	0.706	1	0.985
B4	0	0.404	1	0.222	0	0	0	0.234	0	0
B5	0	0	0	0	0	0	0	0	0	0.015
C1	1	0.394	0.969	1	1	1	0.981	0.029	0	0
C2	0	0.606	0.031	0	0	0	0.019	0.971	0	0
C3	0	0	0	0	0	0	0	0	1	1
D1	1	0.085	0.156	0.944	0.538	0.788	0.673	0.588	0	0
D2	0	0.915	0.844	0.042	0.462	0.212	0.327	0.412	0	0
D3	0	0	0	0	0	0	0	0	1	1
D4	0	0	0	0.014	0	0	0	0	0	0
E1	1	0.170	0.875	0.806	0.500	0.538	0.462	0.794	1	0.970
E2	0	0.830	0.125	0.194	0.500	0.462	0.538	0.206	0	0.030
F1	0.156	0.723	0.141	0.153	0.243	0.173	0.163	0.206	0	0
F2	0.844	0.277	0.859	0.847	0.757	0.827	0.837	0.794	0.906	0.742
F3	0	0	0	0	0	0	0	0	0.094	0.258
G1	1	1	1	1	1	1	1	1	0	0
G3	0	0	0	0	0	0	0	0	1	1

**Table 4.** Frequency of genotypic combinations in different populations of *Ch.* “annularius”. N – the number of individuals, * – original data.

Genotypic combinations	European population	Caucasian populations			Siberian populations			Kazakhstan population	Nearctic populations	
	NL-NT-NT N=16	ECS-BK-ART N=47*	CC-OS-ZM N=32*	SC-SJ- PA N=36*	RU-OMS-IR N=39	RU-NSK-EP N=26	RU-NSK-BE N=52	KZ-AA-BG N=17	US-ND-WA N=16	US-ND-IS N=33
A1.1	0.187	0.574	0.218	0.500	0.820	0.654	0.750	0.529	0	0
A1.2	0.500	0.064	0.188	0.250	0.103	0.193	0.153	0.353	0	0
A2.2	0.313	0.043	0.594	0.028	0	0.115	0.030	0.059	1	0.970
A1.3	0	0.319	0	0.028	0.077	0.038	0.077	0.059	0	0
A1.5	0	0	0	0.139	0	0	0	0	0	0
A2.3	0	0	0	0.055	0	0	0	0	0	0
A2.4	0	0	0	0	0	0	0	0	0	0.030
B1.1	0.687	0	0	0.611	0.103	0.115	0.038	0	0	0
B1.2	0.313	0	0	0	0	0.115	0.135	0	0	0
B1.4	0	0	0	0.333	0	0	0	0	0	0
B2.2	0	0.532	0	0	0.897	0.770	0.827	0.412	1	0.970
B2.4	0	0.128	0	0	0	0	0	0.588	0	0
B4.4	0	0.340	1	0.056	0	0	0	0	0	0
B2.5	0	0	0	0	0	0	0	0	0	0.030
C1.1	1	0.234	0.937	1	1	1	0.961	0	0	0
C1.2	0	0.319	0.063	0	0	0	0.039	0.059	0	0
C2.2	0	0.447	0	0	0	0	0	0.941	0	0
C3.3	0	0	0	0	0	0	0	0	1	1
D1.1	1	0	0	0.889	0.359	0.616	0.481	0.353	0	0
D1.2	0	0.17	0.313	0.083	0.359	0.346	0.385	0.470	0	0
D1.4	0	0	0	0.028	0	0	0	0	0	0
D2.2	0	0.83	0.687	0	0.282	0.038	0.134	0.177	0	0
D3.3	0	0	0	0	0	0	0	0	1	1
E1.1	1	0.043	0.750	0.639	0.256	0.308	0.250	0.706	1	0.940
E1.2	0	0.255	0.250	0.333	0.488	0.461	0.423	0.176	0	0.060
E2.2	0	0.702	0	0.028	0.256	0.231	0.327	0.118	0	0
F1.1	0	0.532	0	0	0	0	0	0.059	0	0
F1.2	0.313	0.383	0,281	0.306	0.487	0.346	0.327	0.294	0	0
F2.2	0.687	0.085	0.719	0.694	0.513	0.654	0.673	0.647	0.813	0.546
F2.3	0	0	0	0	0	0	0	0	0.187	0.394
F3.3	0	0	0	0	0	0	0	0	0	0.060
G1.1	1	1	1	1	1	1	1	1	0	0
G3.3	0	0	0	0	0	0	0	0	1	1

**Arm F** of *Ch.* “annularius” is polymorphic in all the studied populations. In most of them, with the exception of the population from Eastern Ciscaucasia, genotypic combination annF2.2 was predominant (Table 4). In European, two Caucasian (Central and South Caucasus) and all Siberian populations the banding sequence annF1 was present only in the heterozygous state, while in Kazakhstan populations it was found both in homozygous and heterozygous state (Tables 3, 4). As noted earlier, in both Nearctic populations genotypic combination annF2.2 was predominant but also another banding sequence annF3 was present in both homozygous and heterozygous state (Tables 3, 4). Unlike all other populations, in population of Eastern Ciscaucasia the genotypic combination annF1.1 was predominant (Table 4).

**Arm G** is monomorphic in all the studied populations. However, there is an important difference. In Holarctic populations genotypic combination annG1.1 was dominant, while in Nearctic populations another combination annG3.3 was dominant.

The level of inversion polymorphism of Caucasian *Ch.* “annularius” populations is quite similar to those of previously studied Holarctic populations (Table. 5). The populations of the South Caucasus and Eastern Ciscaucasia are generally close to Asian populations (Siberia and Kazakhstan) by all the parameters of chromosomal polymorphism. The population of Central Caucasus is close to the European population by the average number of heterozygous inversions per larvae, number of banding sequences per population and number of genotypic combinations per population. The percentage of heterozygous larvae in population of Central Caucasus is lowest (72%) among all the Holarctic populations (81-90%) (Table 5).

**Table 5.** Cytogenetical characteristics of chromosomal polymorphism in different populations of *Ch.* “annularius”. N – the number of individuals, * – original data.

Cytogenetical characteristics	European population	Caucasian populations			Siberian populations			Kazakhstan population	Nearctic populations	
	NL-NT-NT N=16	ECS-BK-ART N=47*	CC-OS-ZM N=32*	SC-SJ- PA N=36*	RU-OMS-IR N=39	RU-NSK-EP N=26	RU-NSK-BE N=52	KZ-AA-BG N=17	US-ND-WA N=16	US-ND-IS N=33
Heterozygous larvae, %	81	83	72	89	90	85	89	88	18	48
Average number of heterozygous inversions per larvae	1.1	1.6	1.2	1.6	1.6	1.5	1.5	1.0	0.2	0.5
Number of banding sequences per population	10	14	12	15	13	13	14	14	8	11
Number of genotypic combinations per population	11	19	13	19	15	17	18	18	8	12

On the dendrogram of genetic distances, there are four clear clusters that we conditionally assigned as European, Asian, Siberian and Nearctic clusters (Fig. 6). The European cluster is formed by populations of the Netherlands and South Caucasus. The Siberian populations form their own separate cluster and so do Nearctic ones. The populations of Central Caucasus and Kazakhstan form Asian cluster. The population of Eastern Ciscaucasia does not belong to any of the outlined clusters. In spite of relative geographic proximity, the genetic distances between Caucasian populations are quite large (Table 6), and they do not form a single cluster of Caucasian populations. The distance value between populations of Central and South Caucasus (0.3853) does not exceed the distance range (0.136–0.474) for different population of the one species (Gunderina 2001). At the same time, the distance value between populations of Central Caucasus and Eastern Ciscaucasia (0.5318) in one hand and the distance value between populations of Eastern Ciscaucasia and South Caucasus (0.8232) in other hand exceeds those ranges and fall in the distance range (0.474–2.815) for different subspecies (Gunderina 2001). One can see that separation of the population of Eastern Ciscaucasia from other Caucasian populations is relatively big and even reaches a level of subspecies.

**Figure 5.**
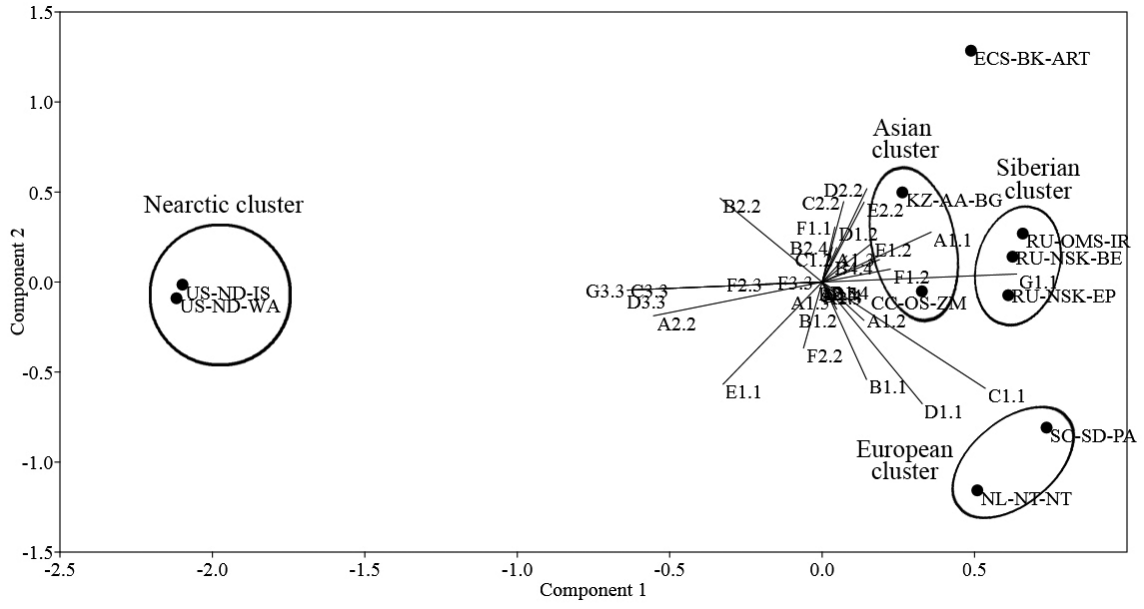
Principal component analysis (PCA) of genotypic combination frequencies in 10 *Ch.* “annularius” populations. For abbreviations of the populations, see Tables 1 and 2.

**Table 6.** Values of genetic distances between the different populations of *Ch.* “annularius”.

Population	NL-NT-NT	ECS-BK-ART	CC-OS-ZM	SC-SJ- PA	RU-OMS-IR	RU-NSK-EP	RU-NSK-BE	KZ-AA-BG	US-ND-WA	US-ND-IS
NL-NT-NT	0									
ECS-BK-ART	1.0628	0								
CC-OS-ZM	0.3919	0.5318	0							
SC-SJ-PA	0.0724	0.8232	0.3853	0						
RU-OMS-IR	0.4069	0.3421	0.4084	0.2454	0					
RU-NSK-EP	0.2589	0.4732	0.3952	0.1566	0.0272	0				
RU-NSK-BE	0.3422	0.3909	0.409	0.2124	0.0147	0.0084	0			
KZ-AA-BG	0.5162	0.4259	0.5787	0.4828	0.4121	0.3845	0.3807	0		
US-ND-WA	1.1784	2.0094	1.1094	1.5183	1.2745	1.1585	1.2025	1.1412	0	
US-ND-IS	1.2873	2.0136	1.2059	1.6637	1.3387	1.2404	1.2917	1.2424	0.0093	0

**Figure 6.**
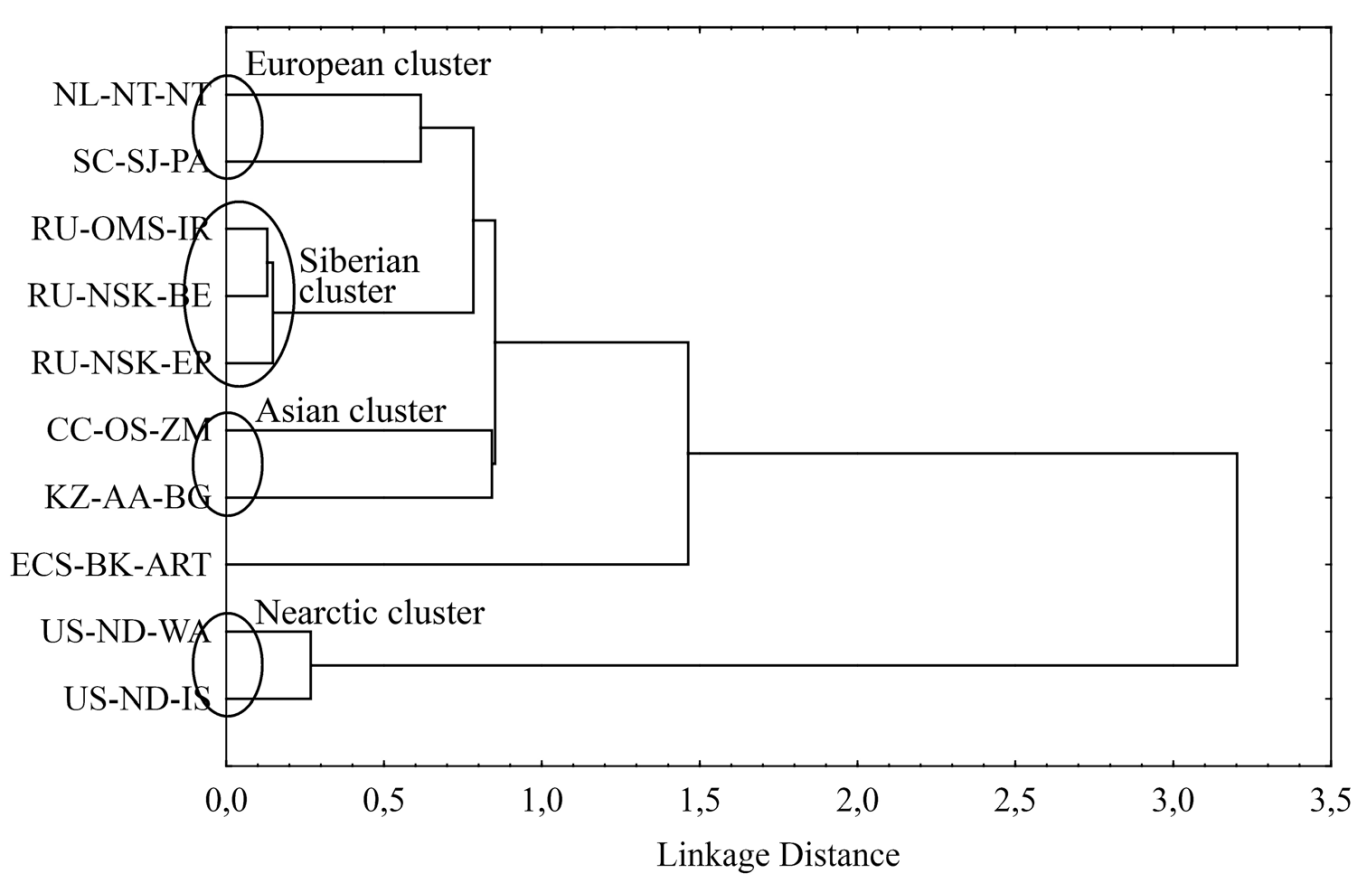
Tree dendrogram for 10 *Ch.* “annularius” populations, *single linkage, Euclidean distances.* For abbreviations of the populations, see Tables 1 and 2.

The principal component analysis shows almost the same picture as the dendrogram of genetic distances (Fig. 6). One can see the dramatic separation of Holarctic and Nearctic populations. In addition, the separation of the European, Asian and Siberian clusters is quite clear. Moreover, the populations of the Nearctic cluster are characterized by a constant increase of genotypic combinations annA2.2, annC3.3, annD3.3, annG3.3, and annF2.3. The European cluster is characterized by increasing of other combinations annB1.1, annC1.1 and annD1.1. The Asian and Siberian clusters are closest to each other. The population of Eastern Ciscaucasia and populations of the European cluster are located on opposite sides of “cloud” of Holarctic populations.

Among Caucasian populations, the frequencies of genotypic combinations in all arms of *Ch.* “annularius” follow Hardy-Weinberg expectation only in the population of South Caucasus. In population of Central Caucasus, the frequencies of genotypic combinations in arm A do not follow Hardy-Weinberg expectation (χ^2^ = 10.166, p – 0.001). The homozygotes annA1.1 were observed 2.22 times more frequently than it was expected and heterozygotes annA1.2 should be occurred 2.29 times more frequently than they were observed. One can observe an even more complex picture in the population of Eastern Ciscaucasia where the frequencies of genotypic combinations do not follow Hardy-Weinberg expectation across three arms: arm A (χ^2^ = 16.046, p – 0.001), arm B (χ^2^ = 25.388, p – 0.000), and arm C (χ^2^ = 5.163, p – 0.023). In the arm A the heterozygotes annA1.2 should be occurred 1.78 times more frequently than they were observed, homozygotes annA2.2 were observed 7.17 times more frequently than it was expected, also expected combinations annA2.3 and annA3.3 were not found at all. In arm B the homozygotes annB2.2 and annB4.4 were observed 1.5/2.1 times more frequently than it was expected and heterozygotes annB2.4 should be occurred 3.77 times more frequently than they were observed. Finally, in arm C the homozygotes annC1.1 and annC2.2 were observed 1.5/1.2 times more frequently than it was expected and heterozygotes annC1.2 should be occurred 1.5 times more frequently than they were observed.

## Discussion

We found the species *Ch.* “annularius” in the South Caucasus for the first time. Earlier (Karmokov 2017) we recorded the species for Eastern Ciscaucasia but without data on its karyotype and chromosomal polymorphism.

Overall, the Caucasian populations of the species can be characterized as relatively polymorphic. We found two new banding sequences annA5 and annD4 in the banding sequences pool of *Ch.* “annularius”. We observed inversion polymorphism almost in all chromosome arms except for arm G, which was monomorphic in Caucasian populations.

Observed picture with Hardy-Weinberg expectation in the site from Eastern Ciscaucasia can be explained in several ways. First, it can be a negative selection of heterozygotes due to some adaptive processes that are still ongoing. Another possibility is that it is due to short time of existence of this population and founder effect.

The climate of Terek-Kuma lowland is much hotter and drier than in both other collection sites. We collected the larvae here from the puddle beside an active artesian well. This habitat is stable because it is constantly fed by water from the well. There are about 3 000 of such kind of wells (most of them still active), within the radius of ca 100 km. Most of them were drilled in the 50–60s of the 20th century for the aims of animal husbandry. Considering this, we can expect a lot of new records of this species from habitats situated beside those wells. The puddle that served as collection site is quite small (3×5m of water surface, max. depth about 0.5m) and thus the total size of the population is not so big. Possibly this population is relatively young and just over 50–60 years old. It can be presumed that initially a very small number of individuals from some nearby habitats established this population and the influx of new migrants is not so large. It is quite possible that most part of the larvae here could be relatives and so the inbreeding could occur quite often. Possibly, there was not enough time for the population to come to the equilibrium. Perhaps we see the founder effect that can also explain the observed picture with Hardy-Weinberg expectation.

All the obtained data are indicative of the complex genetic structure of Caucasian populations of *Ch.* “annularius” and total complexity of microevolution processes occurring in the Caucasus region. In spite of geographic proximity, one Caucasian population is separated from other populations of the Caucasus at the level of subspecies.
